# Sural nerve: imaging anatomy and pathology

**DOI:** 10.1259/bjr.20220336

**Published:** 2022-09-12

**Authors:** Logan Joseph Jackson, Muhamad Serhal, Imran M Omar, Ankur Garg, Julia Michalek, Ali Serhal

**Affiliations:** Department of Radiology, Northwestern University, Chicago, IL, USA; Faculty of Medicine, Lebanese University, Beirut, Lebanon; Department of Radiology, Northwestern University, Chicago, IL, USA; Department of Radiology, Northwestern University, Chicago, IL, USA; Department of Radiology, Northwestern University, Chicago, IL, USA; Department of Radiology, Northwestern University, Chicago, IL, USA

## Abstract

High resolution ultrasound (US) and magnetic resonance (MR) neurography are both imaging modalities that are commonly used for assessing peripheral nerves including the sural nerve (SN). The SN is a cutaneous sensory nerve which innervates the lateral ankle and foot to the base of the fifth metatarsal. It is formed by contributing nerves from the tibial and common peroneal nerves with six patterns and multiple subtypes described in literature. In addition to the SN being a cutaneous sensory nerve, the superficial location enables the nerve to be easily biopsied and harvested for a nerve graft, as well as increasing the susceptibility to traumatic injury. As with any peripheral nerves, pathologies such as peripheral nerve sheath tumors and neuropathies can also affect the SN. By utilizing a high frequency probe in US and high-resolution MR neurography, the SN can be easily identified even with the multiple variations given the standard distal course. US and MRI are also useful in determining pathology of the SN given the specific image findings that are seen with peripheral nerves. In this review, we evaluate the normal imaging anatomy of the SN and discuss common pathologies identified on imaging.

## Introduction

The sural nerve (SN) is an important pure sensory cutaneous nerve which innervates the lateral ankle and foot to the base of the fifth metatarsal. It is formed by contributing nerves from the tibial and common peroneal nerves with multiple different variations described.^1–3^ The SN can be targeted for nerve biopsy and is a common site for autologous nerve grafting.^3–5^ Similar to other nerves in the body, it can develop multiple pathologies including entrapment, contusion, iatrogenic injury, tumors of the nerve and compression from adjacent pathologies.^6–9^ Given the superficial location of the SN and its involvement with peripheral neuropathies, it is a frequent target for diagnostic peripheral nerve biopsies.^4,10,11^ The typical clinical manifestation of the SN pathology is pain and paresthesia along the lateral ankle and foot.^6^ Imaging of the sural nerve is not extensively detailed in the radiology literature; but the SN can be accurately evaluated for abnormalities with both ultrasound (US) and magnetic resonance imaging (MRI). Herein, in this review, we discuss the radiological anatomy and the common pathologies of the SN and their imaging evaluation.

## Anatomy

**Figure 1.**
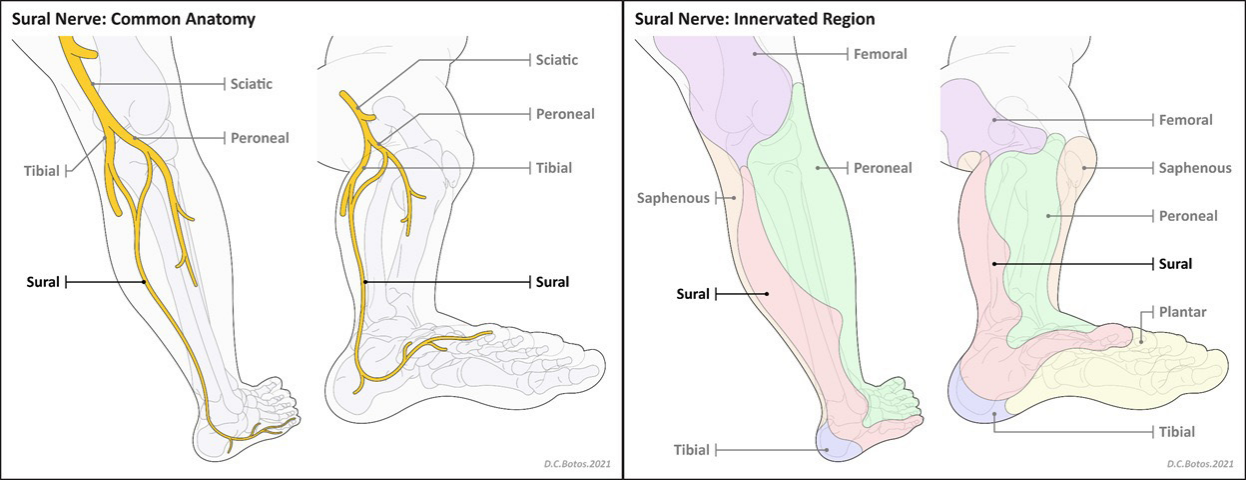
Illustrations showing the course and the origin of the sural nerve from branches of the tibial and common peroneal nerves and the innervated territory of the sural nerve and its components

The SN is a primary sensory nerve that courses down the posteriolateral aspect of the leg to innervate the lateral ankle and foot^12^ (Figure 1). There are six patterns with multiple subtypes describing the formation of the SN in literature with varying prevalence.^3,12^ The most prevalent pattern of the SN origin is the union of two components merging: a medial component arising from the tibial nerve, called the medial sural cutaneous nerve (MSCN) and a lateral component, the peroneal communicating nerve (PCN), arising from the common peroneal nerve or from a branch of the common peroneal nerve, called the lateral sural cutaneous nerve (LSCN).^1,3^ When the SN is formed by the fusion of a medial and lateral component, this typically occurs in the proximal half of the calf.^3^ Other less common patterns include the SN arising directly from the MSCN, directly from the CPN, or directly from the LSCN. There has also been described cases of the SN arising from the sciatic nerve.^12^

The origin of the SN is most commonly subfascial and then perforates the crural fascia as the nerve runs more distally, typically in the mid-calf. There are typical landmark relationships of the SN to surrounding structures that aid in the ability to find the SN radiologically. Proximally, the SN courses between the heads of the gastrocnemius muscles in the posterior knee then more distally, it courses along the lateral aspect of the leg, parallel with the small saphenous vein with varying proximity, as well as having an association with the Achilles tendon..^6,13^ At the level of the ankle, the SN is always present in the lateral retro-malleolar region, separated from the malleolus by the peroneal tendons.^13^ The SN forms the lateral calcaneal branch distal to the lateral malleolus and then courses with the peroneal tendons where it splits into two terminal branches at the level of the fifth metatarsal.^6,14^

## Imaging of the sural nerve

The SN can be evaluated with both US and MRI and these exams can be considered as complimentary. The SN is small with a diameter between 2.7 and 3.4 mm (mean = 3.22 mm);^15^ therefore, its imaging evaluation can be challenging. The anatomic associations of the SN with the small saphenous vein (SSV), the Achilles tendon and the known location in the lateral retro-malleolar region aid in identifying the SN on imaging. US has a higher spatial resolution compared to MRI and can evaluate small details within the SN, while MRI has a better soft tissue resolution and evaluates for nerve increased signal or tumor enhancement. Also, MRI can evaluate the surrounding soft tissue and bone structures for abnormalities involving the SN.

### Ultrasound evaluation of the SN

**Figure 2.**
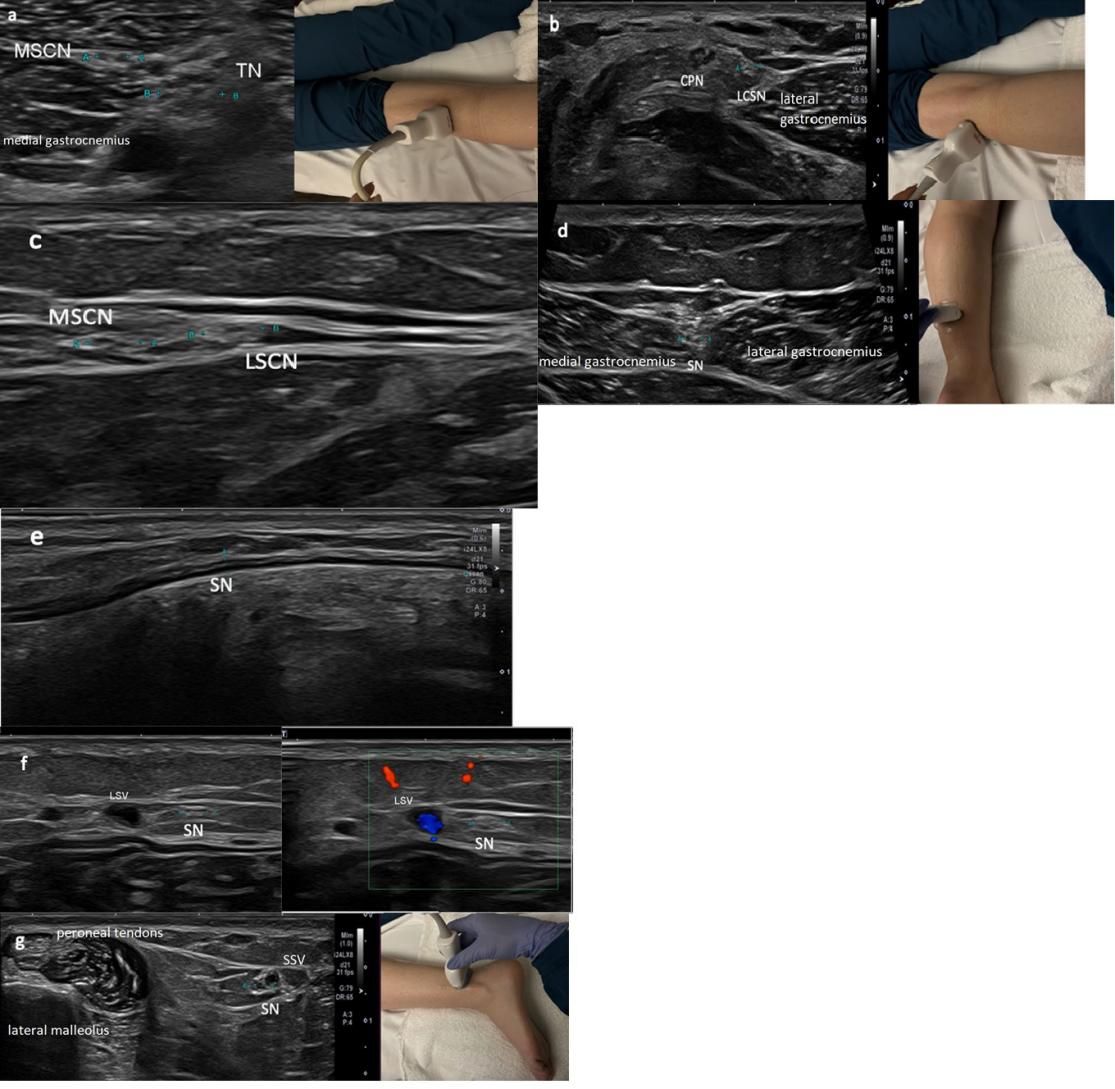
Normal ultrasound image of the sural nerve (SN) in the leg. At the popliteal fossa, image (**a**) shows the origin of the medial sural cutaneous nerve (MSCN) from the tibial nerve (TN) with probe positioning. At the popliteal fossa, image (**b**) shows the origin of the lateral sural cutaneous nerve (LSCN) from the common peroneal nerve (CPN) with probe positioning. Image (**c**) shows the medial and lateral sural cutaneous nerves (MSCN, LSCN) just prior to their fusion in the mid to distal calf and image (**d**) shows the origin of the SN after fusion of the MSCN and LSCN. Image (**e**) is a longitudinal image of the SN in the calf. Image (**f**) shows a transverse gray scale and corresponding color Doppler images at the distal leg level showing the sural nerve (SN) satellite to the small saphenous vein (SSV). Transverse gray scale image (**g**) at the ankle level showing the sural nerve with probe positioning. The SN is posterior to the peroneal tendons and satellite to the SSV

A high-frequency probe should be used for SN evaluation, in our protocol an 18Mhz or 24 Mhz probe is used depending on the patient body habitus. Giving the anatomical variability of the SN at its origin, it is easier to find the nerve distally and to follow it proximally, keeping in mind the different variations previously discussed (Figure 2). The SN is always found in the distal leg adjacent to the SSV and in the lateral retromalleolar area; therefore, it is easier to locate the nerve distally and then follow it proximally into the proximal leg. A normal nerve has a fascicular appearance in transverse section with the small nerve fascicles appear as round hypoechoic structures surrounded by an echogenic background composed of endoneurium, perineurium and interfascicular epineurial fat. The epineurium surrounding the nerve is also echogenic. Long axis imaging of the nerve shows a longitudinal fascicular pattern. The SSV adjacent to it can be easily identified and serves as a landmark to identify the SN. A normal SSV is hypoechoic, compressible and show doppler color. At a variable height, but usually in the proximal third of the leg, the nerve will be separated into two components, the peroneal communicating branch and the medial sural contribution branch, both branches course into the popliteal fossa to join the common peroneal nerve and the tibial nerve, respectively. In the prior section, we described several anatomical variations of the SN origin, which can be found on imaging, where the SN is formed by the continuation of a dominant medial or lateral component originating, respectively, from the tibial or CPN, however, by identifying the nerve distally and following it proximally, these variations should not pose a problem. Pathology of the SN on ultrasound can be similar to other nerves. Neuropathy will present as thickening and hypoechogenicity of the nerve, while a nerve tumor will present as a mass in continuity with the nerve or its branch with increased doppler. Also, nerve transection from trauma will appear as interruption of the nerve.

### MRI evaluation of the SN

**Figure 3.**
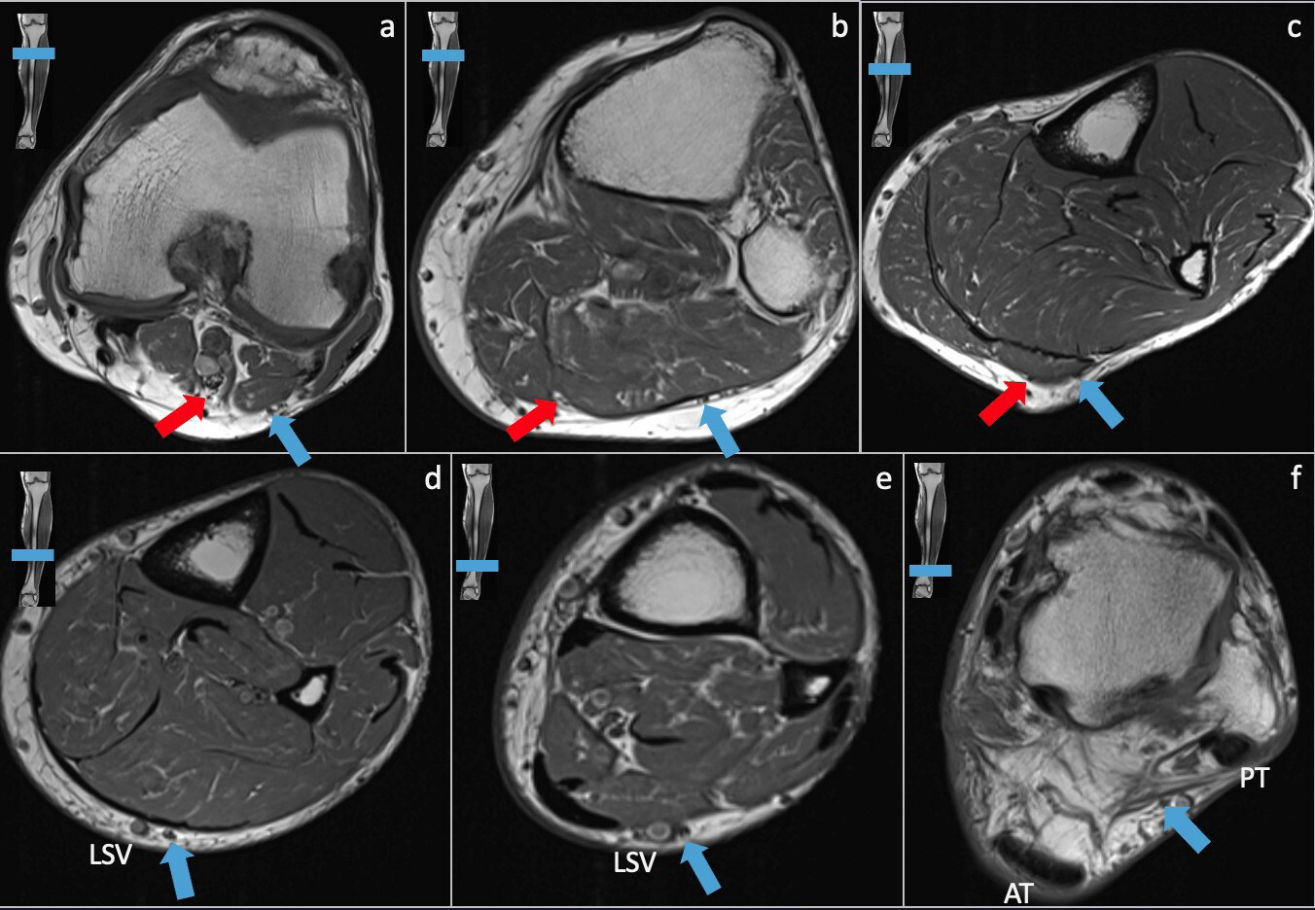
Normal MRI anatomy of the sural nerve. The sural is formed by the fusion of the medial and lateral sural cutaneous branches (arrows in a, (**b, c**) at the mid to distal leg level then courses in the superficial posterolateral soft tissue adjacent the small saphenous vein to the ankle

The SN can be adequately evaluated with high-resolution MRI neurography of the lower extremity. A 3 Tesla magnet is preferred whenever possible to provide higher resolution and signal-to-noise ratio. The MRI protocol, summarized in Table 1, should include a combination of nonfat-suppressed axial thin section sequences, either T1 or proton density weighted and fluid sensitive sequences. A post-contrast 3D isometric STIR sequence reconstructed in multiple planes can be done to suppress the signal from the vessels in the popliteal fossa and the SSV, which makes identification of abnormal signal within the SN easier. It is easier to identify the nerve on axial imaging compared to coronal or sagittal imaging giving its small size (Figure 3). Similar to US evaluation, the nerve can be identified at the distal leg or the ankle level using the anatomical landmark discussed, or it can be identified in the proximal leg by identifying its contributions from the common peroneal and tibial nerves. The MRI appearance of a normal SN is similar to other nerves in the body with fascicular pattern and intermediate signal on all sequence. The fat surrounding the nerve can be used to help in identifying the nerve on nonfat suppressed sequences.

**Table 1.** MR neurography protocol used for upper extremity evaluation. The field of view is adjusted depending on the indication

Sequence	TE	TR	Slice thickness	Flip angle	Acquisition matrix
Axial T1	10	619	3 mm	140	336\0\0\286
Axial T2 FS	104	4000	3 mm	130	256\0\0\230
Coronal STIR	49	4000	3 mm	130	272\0\0\403
Space 3D STIR post contrast	252	3000	Isotropic	variable	0\384\252\0

TE, echo time; TR, repetition time.

## Pathology

Pathology of the SN can be divided into iatrogenic causes, traumatic causes, nerve compression and primary pathologies of the nerve. Sural neuropathy presents as pain and paresthesia of the innervated territory along the posterolateral aspect of the distal third of the leg, the lateral foot and the fifth toe.^14^ The diagnosis is mainly clinical with imaging studies used to confirm the diagnosis and to evaluate for a compression cause that may alter the treatment. The differential diagnosis for sural neuropathy includes sciatica with S1 neuropathy and Achilles tendinopathy. Additionally, in patients with exercise related pain, especially in athletes, the differential diagnoses include exertional compartment syndrome of the lower leg and popliteal entrapment syndrome.^16^

**Figure 4.**
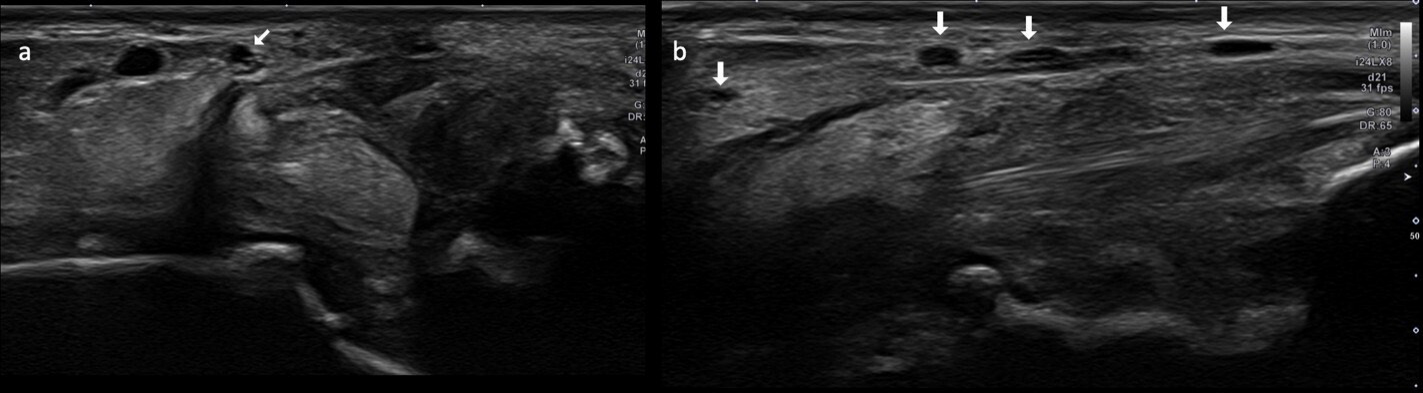
60-year-old female with peroneal brevis tendon rupture and sural neuropathy. Transverse (**a**) and longitudinal (**b**) ultrasound cuts at the level of the ankle shows multifocal nodular thickening of the sural nerve (arrows) without nerve transection consistent with sural neuropathy

**Figure 5.**
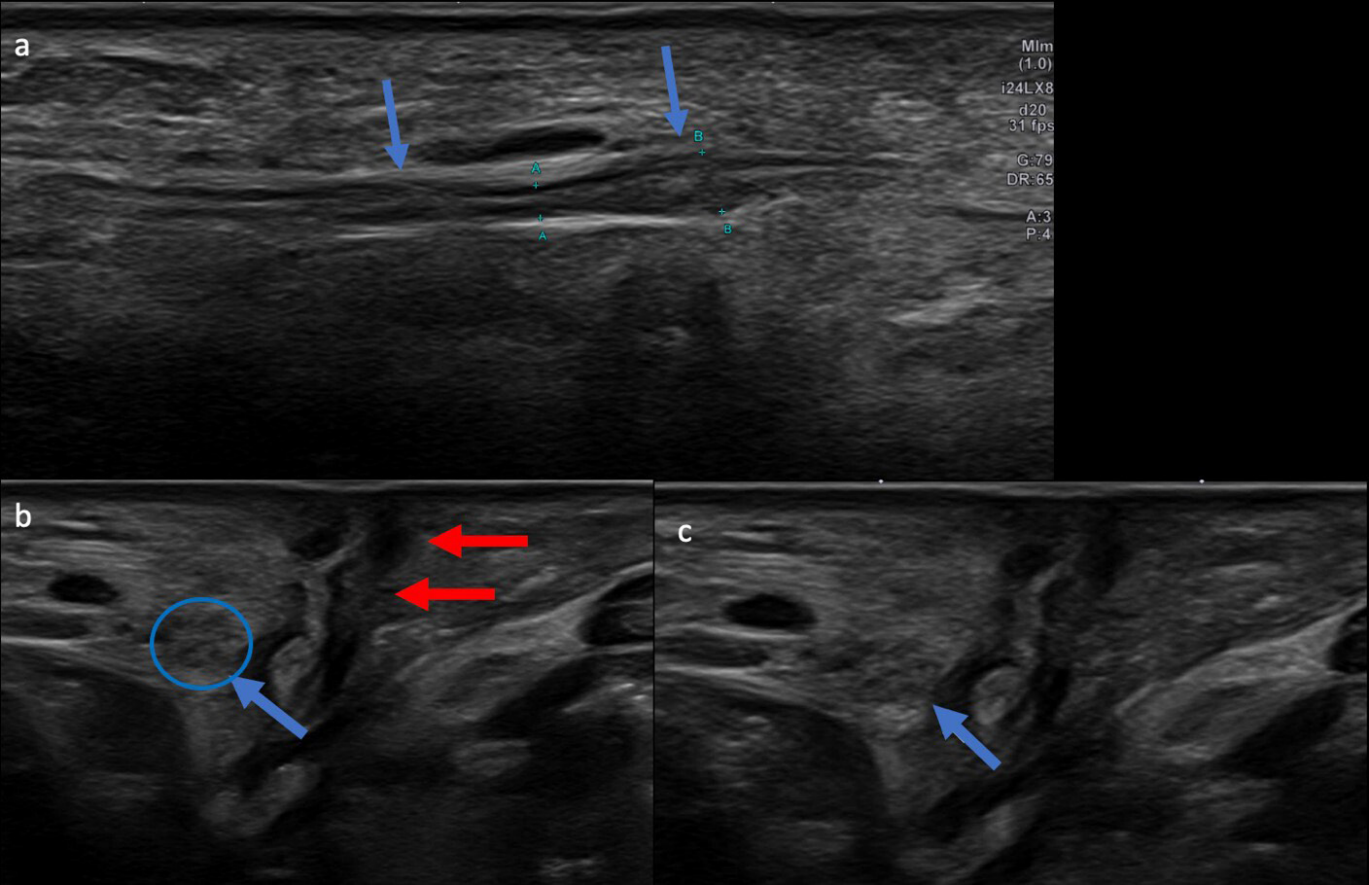
51-year-old male with symptoms of sural neuropathy and prior history of ankle ORIF for ankle fracture. Longitudinal (**a**) and axial (**b, c**) ultrasound cuts at the lateral distal leg level demonstrates focal nodular thickening of the sural nerve at the level of the ankle (blue arrows) adjacent to the soft tissue scar (red arrows) consistent with sural neuropathy without nerve transaction

Giving the close relation of the SN to the gastrocnemius muscles, peroneal tendons, SSV and the Achilles tendon, it is at high risk of injury during procedures that involve these structures^7^ (Figure 4) such as during gastrocnemius recession surgery, Achilles tendon repair, peroneal tendons repair and stripping of the SSV or varices ablation procedures.^6–8^ The most common cause of these iatrogenic injuries during these procedures is due to lack of visualization of the nerve.^8^ Furthermore, SN injury can occur during internal fixation procedures of distal fibular fractures (Figure 5).

Giving the superficial location of the SN in the leg, it is at increased risk of injury during trauma, including closed and penetrating trauma.^6^ Fractures to the calcaneus, lateral malleolus, cuboid or the fifth metatarsal base can also cause direct trauma to the SN.^17^ Furthermore, the SN can be stretched during lateral ankle sprain and can be compressed by a tight cast.

**Figure 6.**
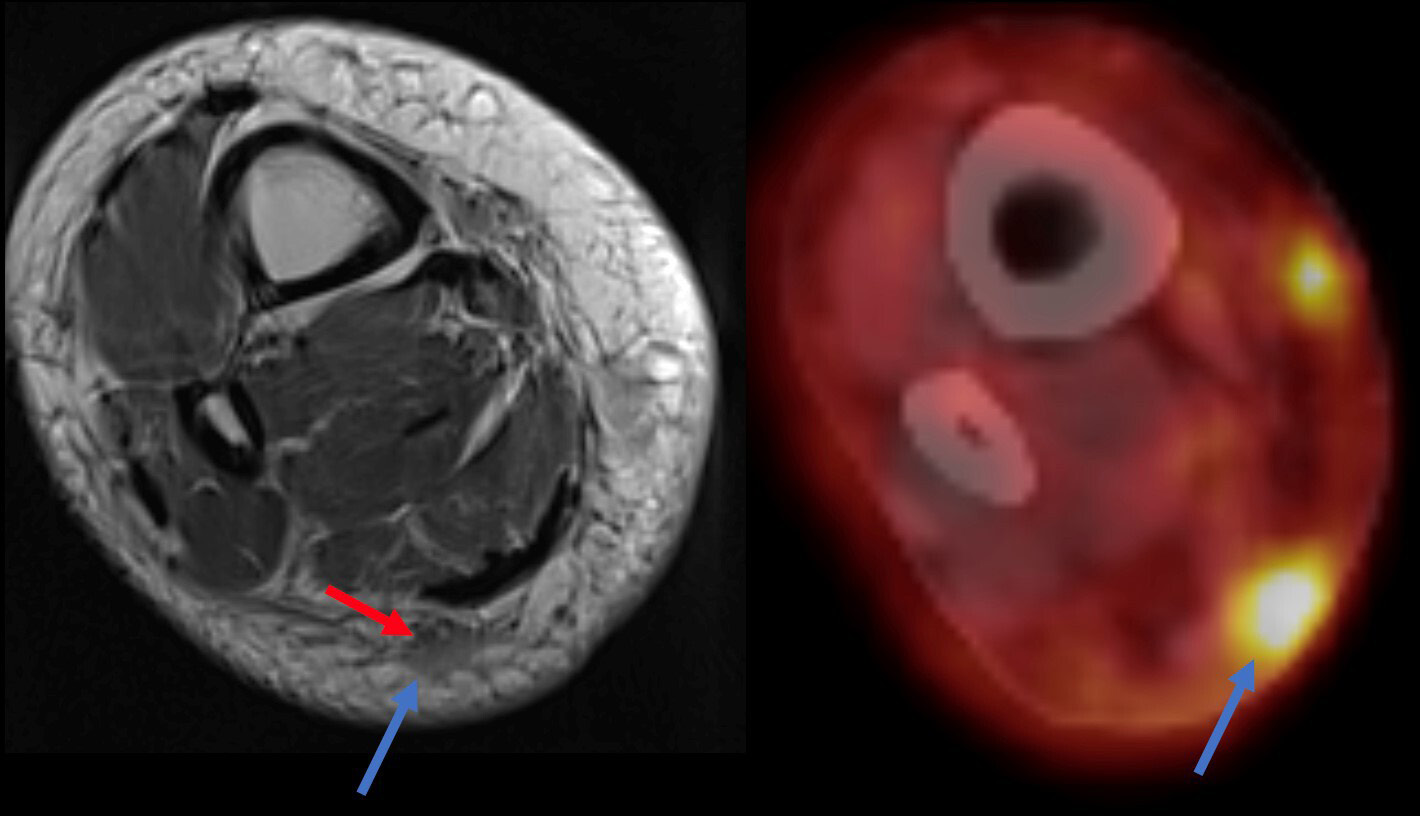
60-year-old female with a biopsy confirmed Merkel cell carcinoma. There is a mass in the posterior subcutaneous fat in the distal calf (blue arrow) that is FDG avid consistent with a tumor deposit. The mass encases the sural nerve (red arrow). The patient had symptoms of sural neuropathy

**Figure 7.**
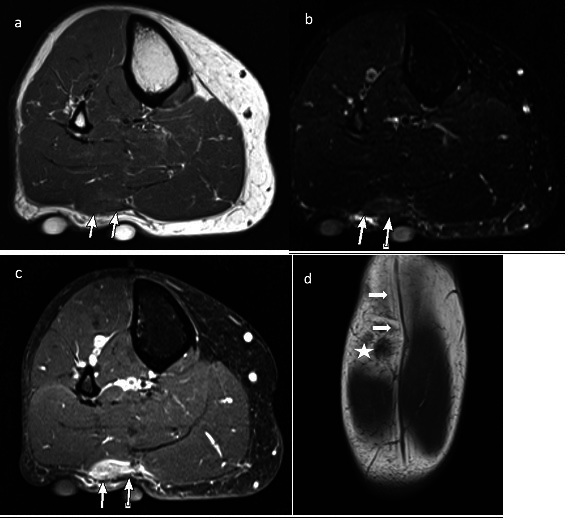
50-year-old female with a palpable mass along the posterolateral mid calf. Axial T1 (**a**) and axial T2 (**b**) weighted images demonstrates a low signal intensity mass (arrows) showing diffuse enhancement (**c**). Coronal *T_1_*-weighted image (**d**) shows the mass (asterix) along the course of the sural nerve (arrow). Pathology demonstrates desmoid fibromatosis

**Figure 8.**
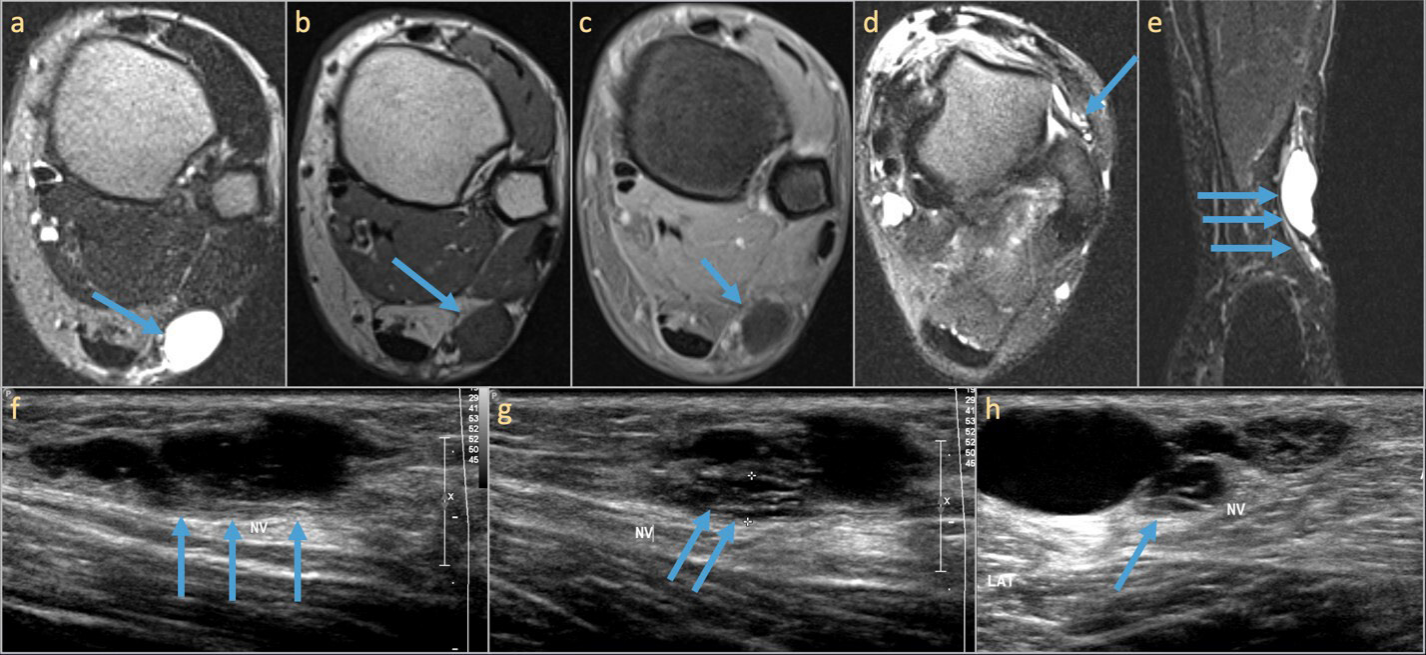
47-year-old female with a palpable mass along the lateral ankle. Axial T2 FS (**a**) shows a cystic high signal lesion abutting the sural nerve (arrow). Axial T1 (**b**), the lesion is low signal abutting and causing mass effect on the sural nerve (arrow). There is no enhancement of the lesion post contrast (**c**). More inferiorly (**d**), the lesion tracks to the anterior ankle joint and is consistent with a ganglion cyst. Coronal STIR (**e**) image shows the lesion and the mass effect on the sural nerve that is slightly hyperintense (arrows). Ultrasound images (**f, g, h**) show the cystic lesion superficial to the sural nerve (arrows). There is thickening and hypo echogenicity of a segment of the sural nerve suggestive of neuropathy

Additional causes of sural neuropathy include nerve compression along its course. The nerve has a long course in the lower extremity from the knee to the ankle joint, including a subfascial course and a more superficial course after piercing the crural fascia. The emergence point of the SN through the crural fascia can be thickened, forming a fibrous arch where the nerve can be compressed, more commonly seen in runners.^17^ Rarely, the medial sural cutaneous branch can have an intramuscular course within the lateral or medial gastrocnemius muscles instead of coursing between the two heads. In these cases, it might be compressed during gastrocnemius contraction, especially in athletes.^18^ Compression of the SN can also occur secondary to space occupying lesions along its course. These can include soft tissue masses, both benign and malignant, such as tumors or metastasis (Figures 6 and 7), or hematomas. Also, ganglia arising from the ankle joints can migrate and compress the SN^6^ (Figure 8).

**Figure 9.**
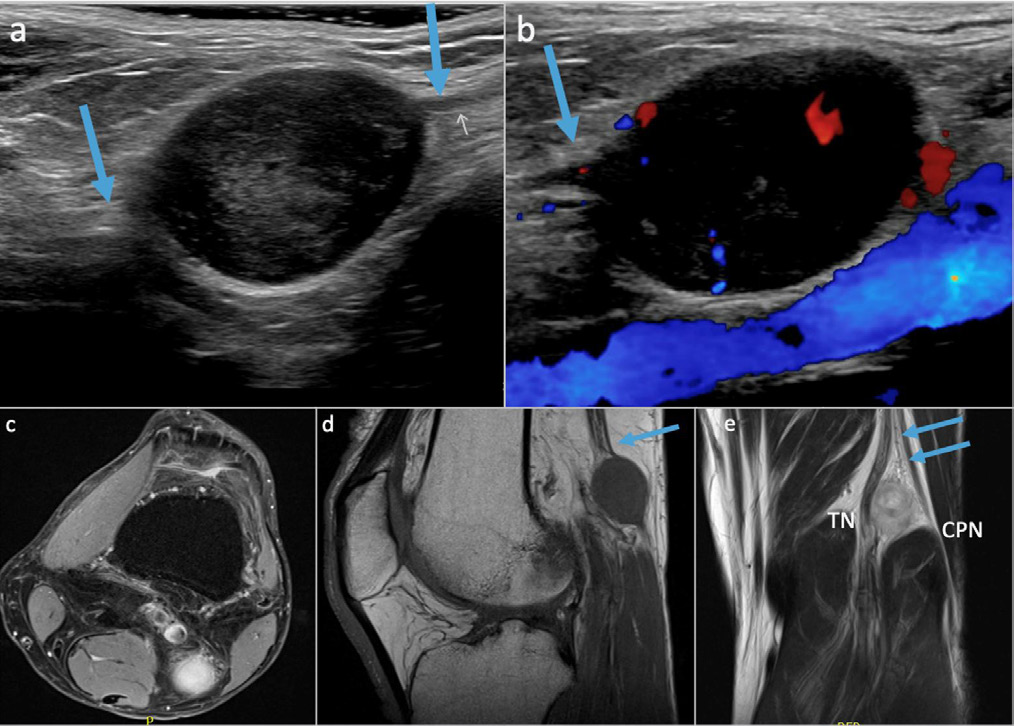
71-year-old male patient with a palpable abnormality in the popliteal fossa. Ultrasound (**a, b**) demonstrates a vascularized ovoid hypoechoic mass that is in communication with a superficial nerve branch (arrows). MRI demonstrates a T2 hyperintense mass lesion splaying the distal sciatic nerve at its bifurcation with mass effect on the common peroneal and tibial nerves origin (**c**). Sagittal T1 and coronal PD images demonstrate the origin of the mass from the lateral sural cutaneous branch (arrows in d and e), a branch from the CPN. Pathology revealed a peripheral nerve sheath tumor

**Figure 10.**
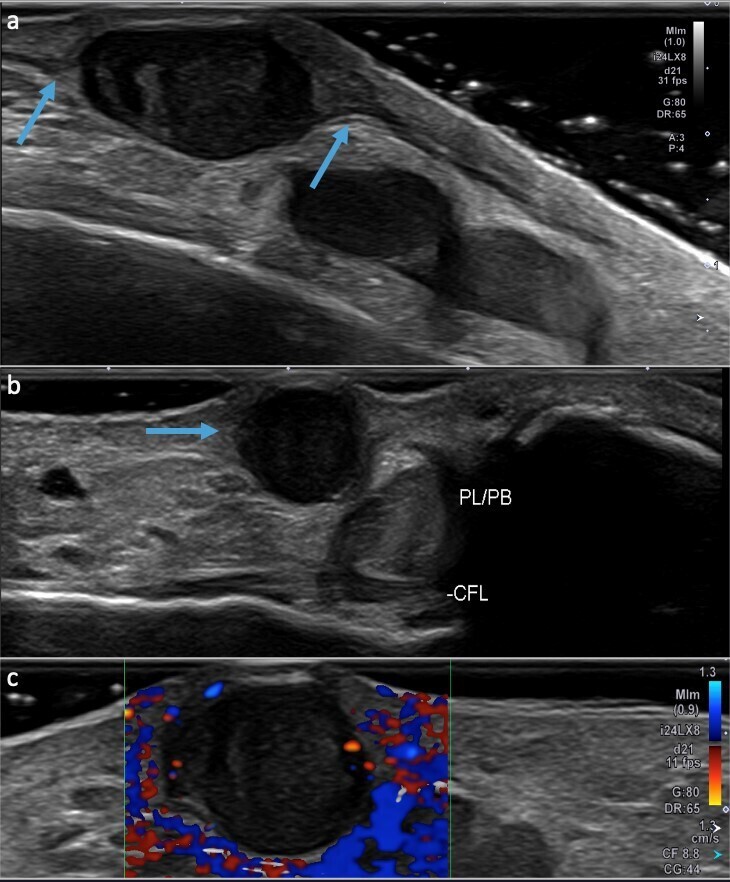
36-year-old female referred for palpable painful lesion in the lateral ankle with a clinical suspicion for a foreign body. Ultrasound demonstrates an oval hypoechoic lesion in continuity with the sural nerve (**a**) and is superficial to the peroneal tendons (**b**). Doppler ultrasound demonstrates mild vascularity of the lesion (**c**). Pathology demonstrates a schwannoma of the sural nerve

Similar to other nerves in the body, peripheral nerve sheath tumors can form in the SN or its branches such as schwannomas^6,9^ (Figures 9 and 10). Neuropathies such as diabetic peripheral neuropathy or familial amyloid polyneuropathy can also involve the SN.^10,11^

## Conclusion

The SN is an important sensory nerve with many anatomic variants that can be utilized for nerve grafting, diagnosing neuropathies, and is associated with numerous pathologies. Knowing the anatomic associations and characteristics associated with specific pathologies enables imaging to be both easy and vital for evaluating the sural nerve. Imaging can also be used whenever procedures are being performed throughout the course of the SN. Given that the leading pathology of the SN is iatrogenic injury, understanding the anatomy prior to any intervention can decrease the incidence of these injuries. Both MRI and ultrasound are complementary in evaluating the SN and should be used to assess both the anatomy and to evaluate for pathology of the SN.
